# Multiple Bound State Soliton Pulses in the All Polarization Maintaining Fiber Laser

**DOI:** 10.3390/mi14081528

**Published:** 2023-07-29

**Authors:** Dalin Sun, Qi Zhao, Shaowen Chu, Chunyu Cao, Jihong Pei, Xintong Xu, Shuangchen Ruan

**Affiliations:** 1 College of Integrated Circuits and Optoelectronic Chips, Shenzhen Technology University, Shenzhen 518118, China; sundalin@sztu.edu.cn (D.S.); 1950416001@stumail.sztu.edu.cn (S.C.); 2210412025@stumail.sztu.edu.cn (C.C.); 2 Shenzhen Technology University Hospital, Shenzhen 518118, China; zhaoqi@sztu.edu.cn; 3 College of Electronics and Information Engineering, Shenzhen University, Shenzhen 518060, China; jhpei@szu.edu.cn; 4 College of New Materials and New Energies, Shenzhen Technology University, Shenzhen 518118, China

**Keywords:** zeolite, polarization maintaining, fiber laser, bound state soliton, mode locked

## Abstract

The bound state soliton pulse, a novel mode-locked output state of fiber lasers, has been studied extensively to gain a better understanding of soliton interactions and to explain the mechanism behind the generation of mode-locked pulses. In this particular research, we utilized a self-made saturable absorber (SA) consisting of single-walled carbon nanotubes (SWCNT) in a fully polarization maintaining (PM) erbium-doped fiber optical path. Through this setup, we observed various bound state pulse phenomena, including the double bound state with different phase differences, the bound state formed by two double pulse bound states, the multi-pulse bound state, etc. The abundant bound soliton pulse states demonstrated the excellent nonlinear absorption characteristics of the SA as well as the excellent optical properties of the all-PM fiber laser. It contributed to exploring the relationship between sub pulses and mode-locked pulses in the future. Additionally, due to the strong interaction between bound state solitons and the inherent stability of the PM optical path, there was potential for utilizing this setup as a seed source to enhance the stability of high-power fiber lasers.

## 1. Introduction

Bound state soliton pulses, also known as soliton molecular pulses, were two or more sub pulses that were bound together through the interaction between pulses. These sub pulses were attracted to each other when they were far apart and repelled when they were close together. As a result, they reached stable-state equilibrium, forming soliton molecules with the same energy and pulse width, thus creating a pulse [1,2,3]. The strength of the interaction between the pulses determined whether they formed tight bound state solitons or weak bound state solitons. The soliton spacing of tightly bound solitons was small enough, and through nonlinear direct action, the spacing between pulses was fixed, and the phase difference could only be discrete values such as 0, −π/2, π/2, π [4,5,6,7]. When the soliton spacing in the bound state pulse was relatively large, it was called the weakly bound state soliton and could only indirectly interact with continuous waves and dispersive waves [8,9,10]. This weak bound state soliton pulse had unstable pulse spacing with no fixed phase difference, was vulnerable to environmental interference, and became unstable [11]. In our experiment, we observed multiple bound state soliton pulses that exhibited tight binding and possessed strong anti-interference capabilities. These tightly bound states demonstrated remarkable stability, as slight interference did not disrupt the pulse balance. This characteristic of strong stability and resistance to interference enhanced the research value of our findings.

The research on bound state pulses has been developed for many years, from theory to experiment. In 1993, Malomed et al. theoretically proved that the bound state pulse had stable soliton spacing and phase difference (0 or π) [5]. In 1997, N Akhmediev and others confirmed the existence of π/2 phase difference bound state pulse [12]. Only after 2002 did Grelu and Belhache et al. observe the bound state pulse with a phase difference of ± π/2 through experiments [2,13]. The phase difference relationship of the bound state mode-locked sub pulse could be judged by its spectral shape. As the phase difference *θ* = 0 or π, the spectral shape was distributed symmetrically around the central wavelength: *θ* = 0 (referred to as in-phase), the pulse intensity at the center wavelength was the highest; *θ* = π (known as inversion), the pulse intensity at the center wavelength was the smallest. When the phase difference *θ* was π/2 or −π/2, the spectral shape was asymmetric: *θ* = π/2, the second peak of the spectrum underwent a red shift to the right of the maximum peak of the central wavelength; *θ* = −π/2, the second peak of the spectrum underwent a blue shift to the left of the maximum peak of the central wavelength. In addition to the double bound state pulse, the triple pulse bound state soliton was also experimentally confirmed subsequently [14]. The spectrum showed periodic modulation, and the pulse time interval was fixed. In the development process, people found that the most important phenomenon was that the spectral modulation (Δλ) of the bound state mode-locked pulse and the time interval (ΔT) of the sub pulse satisfied the formula:

(1) $$∆T=\frac{\lambda_{0}^{2}}{c\cdot ∆\lambda }$$

where *λ*_0_ was the spectral center wavelength and *c* was the speed of light in a vacuum, taken as 3 × 10^8^ m/s.

So far, there have been two main ways to obtain bound state pulses, including active modulation [15] and passive modulation. Compared to the active scheme, the passive modulation was simpler, more flexible, and cheaper, thus having better applicability. Including nonlinear optical loop mirror (NOLM) as artificial saturable absorbers (SAs) and graphene, MoTe_2_, MoS_2_, and carbon nanotube [16,17,18,19,20] as real SAs. As for these investigations, one closely related issue was how to switch the produced bound states in a fiber laser from one to another reproducibly. It was noted that in the previous work, almost all bound states required adjustment of pump power and polarization states in the cavity. Moreover, the bound state pulses obtained in this way often acquired only one state or were accompanied by a tunable center wavelength [21]. In recent years, extensive research has been conducted on photonic crystals [22,23,24], and the tunability of lasers has been successfully achieved by using SWCNT [25]. Inspired by these significant advancements, our study aimed to explore the potential of combining zeolite photonic crystals with carbon nanotubes. Notably, our findings demonstrated, for the first time, the observation of more than three tunable multiple pulse states within a cavity characterized by an invariant polarization state.

In this work, we used porous zeolite (structure code AFI) as the host material to synthesize carbon nanotubes through the pyrolysis template method and assemble them into SWCNT@AFI SA, following the same procedure as reported in [26,27,28]. We had found that this homemade SA had the ability to obtain Q-switched and mode-locked pulses earlier, but this was the first time that we observed the bound state soliton pulse phenomenon in an erbium-doped optical path, which further expanded its application field. Since the bound state pulses, we obtained were all tight bound state soliton forms. In order to make the laser have stronger anti-environmental interference ability, we used a full PM formation and eliminated the need for polarization controllers (PC). Therefore, we only need to adjust the pump power to acquire different tight bound state soliton pulses. Compared with other traditional single-mode fiber optical paths with extra modulation devices, such as PCs, the output pulses could maintain longer stability. This was the highlight of this work and also laid the foundation for the industrial transformation of laboratory achievements.

## 2. Materials, Methods and Characterization

### 2.1. SWCNT@AFI SA

**Figure 1:**
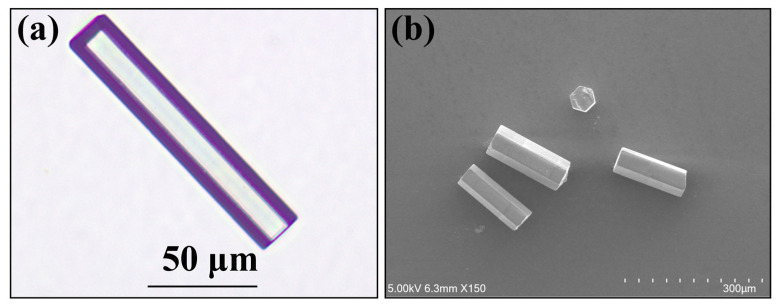
(**a**) The optical morphology image of the transparent AFI crystal; (**b**) The SEM images of the SWCNT@AFI SA.

The SWCNT@AFI SA used in this work was obtained following the same steps as what we have previously reported [29,30,31]. The zeolite single crystal (ZnAPO-5, framework type code: AFI) was fabricated by hydrothermal synthesis. An initial gel with a composition of 1.6Al_2_O_3_: 1.2P_2_O_5_: 0.2ZnO: 1.2TPA: 1.0HF: 600H_2_O was stirred and sealed into the Teflon-lined autoclave and heated to 438 K for 20 h for the pure AFI single crystal. And then the as-synthesized crystals were heated from room temperature to 873 K in one and a half hours in a vacuum crucible furnace of 10^−3^ mbar for two hours to form the SWCNT@AFI SA. The parallel open channels of AFI and the doped Zn^2+^ metal ions were beneficial for the stability and density of the SWCNT. Figure 1 shows the optical morphology and SEM images of the pure AFI crystals (a) and the SA (b), respectively.

**Figure 2:**
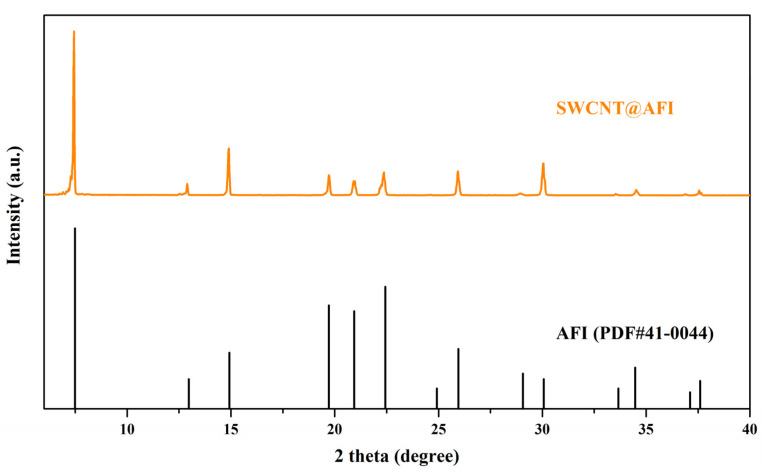
This is the XRD pattern of the as-synthesized SWCNT@AFI SA and the standard card of AFI in the database of zeolite structures.

**Figure 3:**
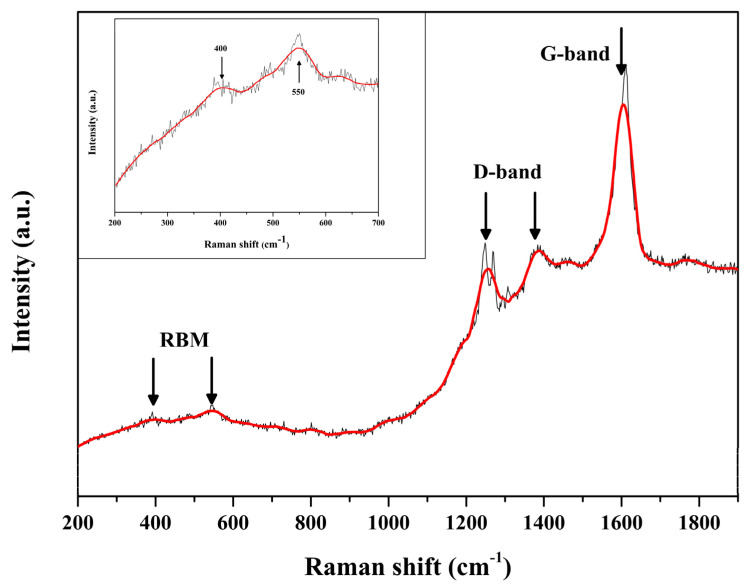
The Raman spectral fitting data and local maps of synthetic carbon nanotubes.

In addition to characterizing the crystal morphology by using microscopes, we also used X-ray diffraction and Raman spectroscopy to analyze the phase composition of the crystal and the synthesis quality of carbon materials. Figure 2 shows the XRD results of the crystal after carbonization. Compared with the standard card AFI (PDF#41-0044) in the zeolite database, the characteristic peaks of the synthesized SWCNT@AFI crystals were completely consistent, with values of 7.43°, 12.89°, 14.89°, 19.74°, 20.97°, 22.40°, 25.92°, 28.93°, and 30.02°, respectively. It proved that the crystal had a standard AFI structure, a single component, good crystallinity, and no impurities after carbonization, indicating that the crystal had not collapsed. There were many methods to characterize carbon materials [32,33,34,35]. Among them, the advantage of Raman spectroscopy was that it could provide sufficient information on the structure of carbon materials while ensuring that the zeolite pore structure and single-walled carbon nanotubes were not damaged. We also adopted this method for our measurements in this section. Typically, for the carbon materials, the Raman spectroscopy would exhibit three typical oscillation modes or bands [36]: (a) The low frequency band 400–600 cm^−1^ called the radial breathing mode (RBM); (b) The medium frequency band 1200–1500 cm^−1^ called the disordered carbon band (D-band); (c) The high frequency band 1500–1620 cm^−1^ called the graphitic carbon band (G-band). As depicted in Figure 3, the Raman spectrum of the carbonized crystal exhibited a strong G-band, indicating a high degree of graphitization of carbon. We emphasized the information of the RBM band in the local image, as this region represented the chirality of carbon nanotubes, and the peaks of 400 cm^−1^ and 550 cm^−1^ indicate that the SWCNT we synthesized were (4,2) chiral [37,38,39].

Finally, through synthesis and characterization, it was confirmed that we had successfully synthesized SWCNT@AFI SA. The main modulation role in the experiment was played by the nonlinear absorption characteristics of SWCNT. Meanwhile, the host zeolite material served to raise the threshold of photothermal damage and prevent the aggregation of nanomaterials [40,41,42]. It was worth noting that the self-made SA we were referring to was one single crystal rather than several crystals in a matrix.

**Figure 4:**
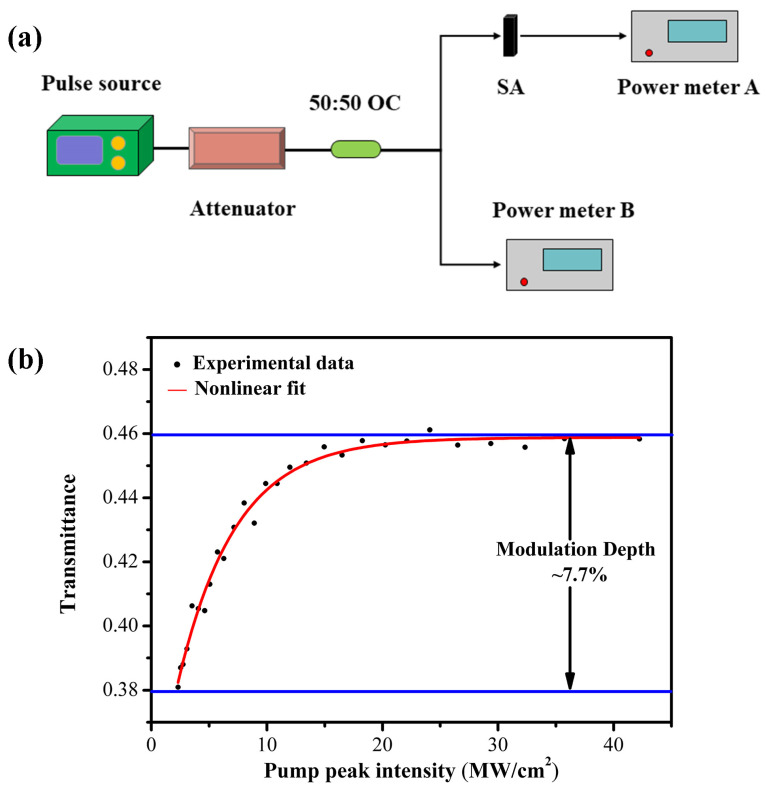
(**a**) A schematic diagram of the dual balance detector system; (**b**) The typical normalized saturable absorption curve of the SA, with an excitation wavelength of 1556 nm.

Finally, a twin-balanced detector, as illustrated in Figure 4a, was used to characterize the nonlinear optical response of the SWCNT@AFI SA, of which the pulse source was a picosecond pulsed fiber laser with a central wavelength of 1556 nm. Connected to a 50:50 fiber optical coupler (OC) through an attenuator, and the attenuator included a fiber isolator to ensure unidirectional laser transmission. One end of the coupler was directly connected to the power meter B, and the other end was connected to the power meter A after coupling SA with a pair of optical fiber jumpers. The black dots in Figure 4b represented the experimental data converted into power density, which conformed to the trend of nonlinear absorption, and the red solid line represented the fitting curve of the experimental data. According to the saturable absorption model [43]:

(2) $$\alpha \left(I\right)=\alpha_{ns}+\frac{\alpha_{0}}{1+\left(\frac{I}{I_{sat}}\right)}$$

where *α*(*I*) represents nonlinear saturable absorption characteristics related to SA and incident light power density; *α_ns_* the non-saturable loss; *α*_0_ the modulation depth; *I* incident light intensity; and *I_sat_* the saturation intensity. The modulation depth was approximately 7.7%, indicating the presence of typical saturated absorption effects.

### 2.2. ALL-PM Fiber Laser

Currently, much of the research on ultra-short pulses is primarily conducted in laboratory settings, which limits their practical applicability in complex and dynamic working environments such as factories, wilderness areas, oceans, and space. Efforts were being made to develop robust and compact ultra-short pulse devices that could withstand challenging environments and meet the requirements of different industries. In order to convert laboratory achievements into practical applications, we adopted a fully polarized optical path structure in this experiment. The special physical structure of polarization maintaining fiber optic devices (stress columns) could enhance the birefringence effect of the fiber, better resist external stress interference, and thus adapt to complex environmental interferences such as vibration, air pressure, temperature, humidity changes [30,44], etc.

**Figure 5:**
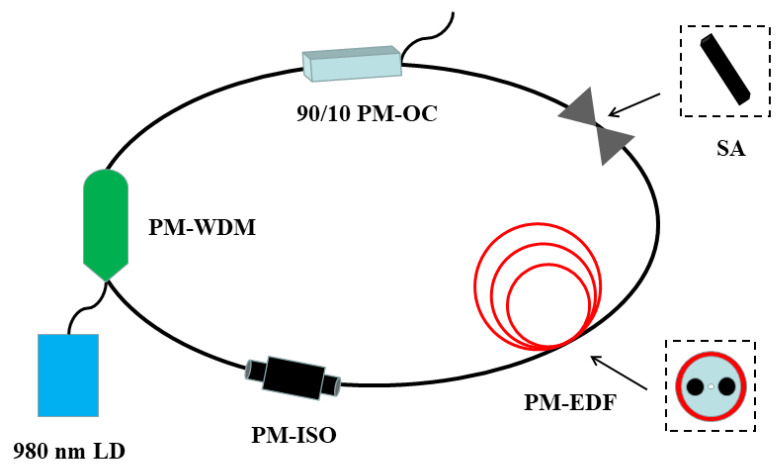
Schematic of the experimental setup for the all-PM fiber laser.

The all-PM fiber laser was shown in Figure 5. A piece of 1.2 m Er-doped fiber (Nufern PM-ESF-7/125) with a core absorption of ~24.0 dB/m was used as the gain medium. The whole optical path was excited by a PM 976 nm continuous laser diode, and wavelength division multiplexing (WDM) was used to combine optical signals of different wavelengths and couple them into the same fiber for laser transmission. There was also an isolator (ISO) with its fast axis blocked in the fiber laser to ensure unidirectional laser transmission. However, we did not install the polarization controller, so the linear polarization state of the laser should be maintained. Finally, the self-made SA was fixed by a pair of FC/APC connectors and then inserted into the optical cavity by a PM optical coupler (PM-OC) with a 90:10 coupling ratio. The polarized light was directed onto the SA via the connector, allowing it to pass directly through the SA. The light then exited through another connector, ensuring a complete fiber laser ring cavity and enabling laser modulation based on the saturable absorption characteristics of the SA. The temporal characteristics of the export laser were monitored using a combination of a 4 GHz high-speed oscilloscope and a 15 GHz bandwidth battery biased fiber optic detector (Newport 818-BB-35F).

## 3. Results and Discussion

**Figure 6:**
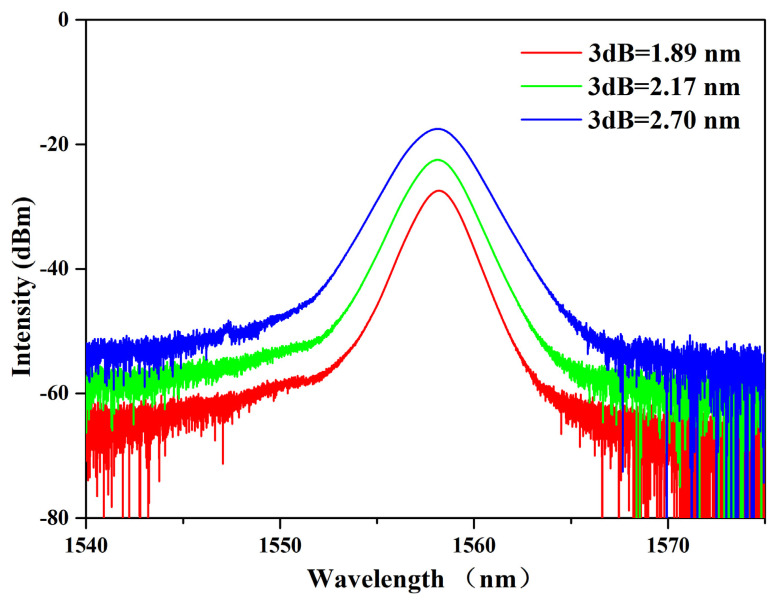
Schematic diagram of the spectral variation trend of traditional soliton mode-locked.

The length of the gain fiber was not predetermined in this experiment. In fact, due to the relatively large gain coefficient of the gain fiber, we first used a length of 0.85 m PM erbium-doped fiber and obtained the traditional soliton mode-locked spectrum shown in Figure 6 at a pump power of 220 mW. At the initial power of 220 mW, we obtained the spectrum shown as the red curve in Figure 6, with a central wavelength of 1558.19 nm and a 3 dB bandwidth of 1.89 nm. As we increased the pump power to 320 mW, the spectrum exhibited the green line, with a slight blue shift in the center wavelength of 1558.12 nm and a 3 dB bandwidth of 2.17 nm. Finally, when the threshold power of 400 mW was reached, the spectrum presented the blue curve and further blue shift, with a central wavelength of 1558.09 nm and a 3 dB bandwidth of 2.70 nm. During the process of spectral broadening, only the pump power increased, while the cavity length remained unchanged at 6.7 m, accompanied by a gain fiber (PM-ESF) length of 0.85 m. And we found that as the pump power increased, the oscilloscope exhibited a trend of pulse jitter and splitting. Instead of making the pulse stable, we further lengthened the length of the gain fiber to provide more gain media to increase the jitter of the pulse in order to obtain new mode locking phenomena.

**Figure 7:**
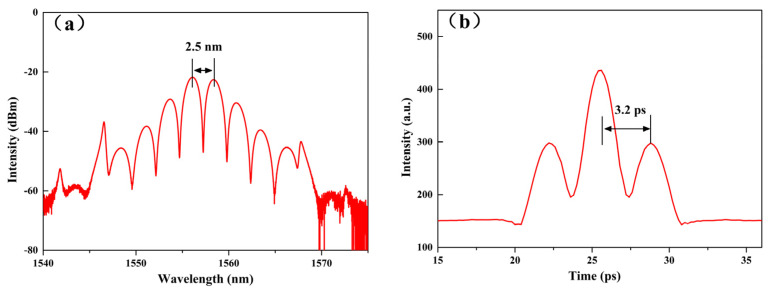
The bound state mode-locked pulse of the all-PM erbium-doped fiber laser: (**a**) Emission spectrum; (**b**) Pulse autocorrelation curve.

Subsequently, when the PM-ESF increased to 1.2 m, we obtain a stable bound state soliton spectrum for the first time, as shown in Figure 7. It was well known that optical solitons, as locally stable nonlinear waveforms, were obtained by striking a balance between fiber dispersion and nonlinear effects [45]. When several solitons coexisted at relatively small intervals in the laser cavity, they evolved into bound solitons, also known as soliton molecules. The dynamic balance between nonlinearity, dispersion, and energy exchange brought about by the growth of gain fibers enabled the conversion of traditional solitons to bound solitons [46,47,48,49,50,51]. This tight bound state was formed mainly due to the direct interaction between closely spaced pulses [52]. Right now, the pump power is 165 mW, showing a stable double bound state mode-locked pulse. As we could see from Figure 7a, the spectral shape was distributed symmetrically around the central wavelength of 1556.80 nm, so it was determined that the phase difference was 0 or π. Then, since the peak intensity was weakest at the central wavelength, we judged it to be an inversion bound soliton with a phase difference *θ* = π. Figure 7b shows the autocorrelation curve of the obtained double bound state pulse, which contained three equidistant peaks and a peak intensity ratio of 1:2:1. It indicated that the binding force between the sub pulses was strong and that the two soliton pulses had the same intensity and pulse width. Moreover, the central wavelength of the pulse *λ*_0_ = 1556.80 nm, spectral modulation Δ*λ* = 2.5 nm, and time-domain interval ΔT = 3.2 ps, satisfied the equation stated in Equation (1).

As we continued to elevate the pump power, we found that more different forms of bound state mode-locked pulses could be observed in this stable PM optical path, including the typical spectral shapes. It was noted that all of these bound state solitons could operate stably in the power range from 165 mW to 342 mW.

**Figure 8:**
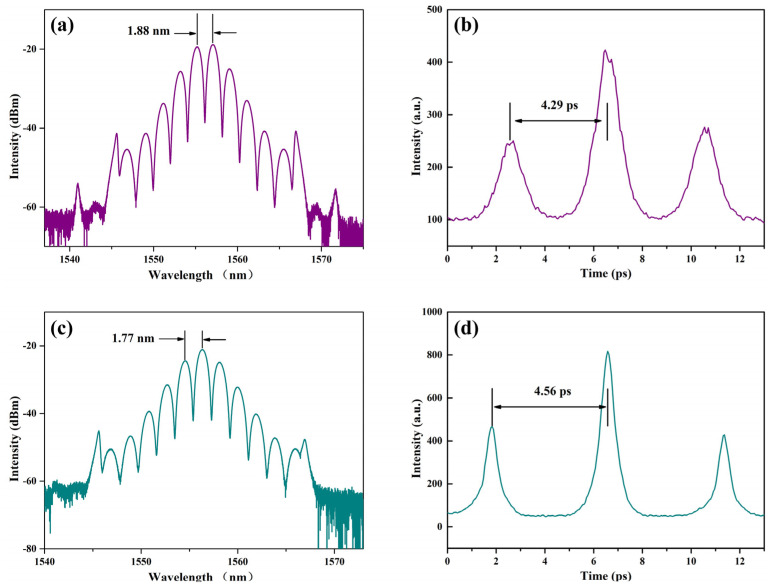
Bound state pulses of the all-PM erbium-doped fiber laser based on SWCNT@AFI SA: (**a**) Emission spectrum with a phase difference of π; (**b**) The pulse autocorrelation curve with a time interval of 4.29 ps; (**c**) Emission spectrum with a phase difference of 0; (**d**) The pulse autocorrelation curve with a time interval of 4.56 ps.

Figure 8a was another bound state soliton emission spectrum with a sub pulse phase difference of π at a pump power of 205 mW, and the central wavelength changed a little, *λ*_0_ = 1556.11 nm. Compared with the bound state pulse in Figure 7, the difference was that the pulse spectral modulation period was shortened, Δ*λ* = 1.88 nm, and the calculated time interval, ΔT = 4.29 ps, was consistent with the measurement result, as shown in Figure 8b. It had also been proven that a smaller modulation period corresponded to a larger soliton time-domain spacing. At 238 mW pump power, a bound state soliton emission spectrum with a sub pulse phase difference of 0 was shown in Figure 8c. In this state, the peak at the central wavelength was the highest, and the center wavelength shifted slightly to λ_0_ = 1556.36 nm, the spectral modulation period to Δ*λ* = 1.77 nm, and the calculated time interval to ΔT = 4.56 ps (shown in Figure 8d). This indicated that the bound state solitons with different phase differences could be switched by adjusting the excitation energy. From the above series of double soliton bound state pulses, we could flexibly control the switching of bound state pulses by adjusting the pump power. This was due to the fact that when the pump power in the fiber cavity changed, the soliton pulse energy rose above the threshold and the nonlinear effect increased, resulting in the original soliton dispersion and the generation of different bound states [53]. At the same time, when the bound state soliton is switched, nonlinear effects and dispersion need to be balanced in the multimode interference range [54].

**Figure 9:**
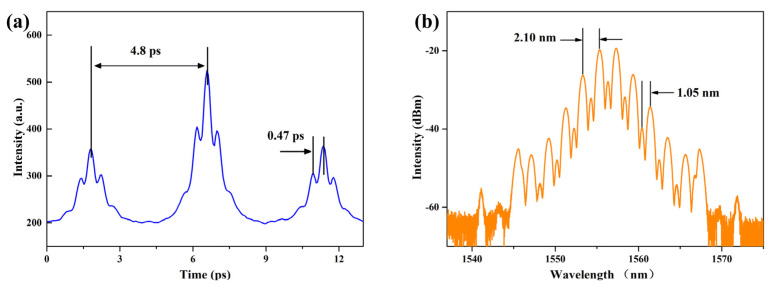
(**a**) Bound state autocorrelation of the double pulse bound state soliton’s bound state; (**b**) Emission spectrum of multi-pulse bound state solitons.

Different from the previous double soliton bound states, by continuing to increase the pump power, we got the two special bound states shown in Figure 9. Figure 9a was the double pulse bound state soliton’s bound state at a pump power of 281 mW, which was better displayed on the autocorrelation curve. It was the bound state formed by two double pulse bound states. In this state, the double bound state pulse soliton as a soliton primitive generated a new bound state. At first, every two bound solitons combined together to form a bound group, and then the bound group was further combined as a unit into other bound groups in the same way [55]. So, this bound state contained two different time intervals, which were respectively the double bound state soliton’s ΔT_1_ = 0.47 ps and the new bound state soliton primitive ΔT_2_ = 4.8 ps. According to the emission spectrum, there should also be two spectra with modulation periods of 17.17 nm and 1.68 nm, respectively. Our results showed that the primary bound solitons could also function as a unit to form a new bound state. In addition, these two bound states in this condition were both stable, and they could last several hours if no disturbance was introduced after obtaining them.

Finally, Figure 9b was the emission spectrum of a multi-pulse bound state soliton pulse in the power range of 300–324 mW, which referred to the whole soliton composed of three or more multiple equally spaced pulses with the same pulse intensity and pulse width. The multi soliton bound state, formed by the interaction between solitons, comes from the further splitting of soliton molecules. When the pump power was elevated large enough, due to the peak power clamping effect and the soliton energy quantization effect, several solitons would be generated in the laser cavity, and then these solitons interacted to form multiple bound state solitons [56]. Taking the four pulse bound state soliton as an example, its peak intensity ratio should be 1:2:3:4:3:2:1 [55], and the phase difference also meets the relationship with the spectral shape. Compared with the double pulse bound state soliton, the modulation in the multi-pulse bound state soliton was no longer single, but the number of sub pulses needed to be observed through the autocorrelation curve [57]. Therefore, limited by experimental conditions, in Figure 9b, we could determine that a multi-pulse bound state had occurred, but we could not judge the specific number of solitons.

**Figure 10:**
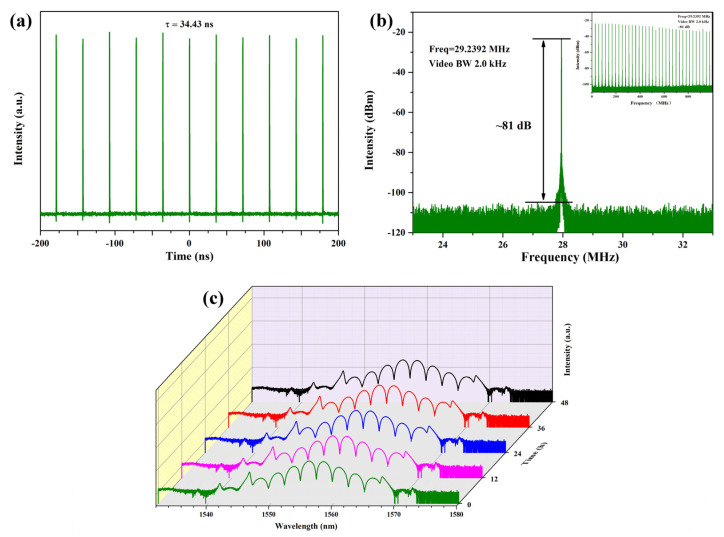
Output characteristics in the bound state mode-locked state: (**a**) Output pulse train of the all-PM erbium-doped fiber laser; (**b**) The RF spectrum measured around the repetition rate of 29.24 MHz, inset: RF spectrum in a 1 GHz span; (**c**) The evolution of spectra on time (0-48 h; interval was 12 h).

Thus, it could be seen that the fully polarized optical path we had built had the ability to achieve rich and diverse bound state soliton pulses and only required a simple adjustment of pump power. This ability was attributed to the exceptional modulation performance of the SA, which further confirmed the advanced nature of our self-made SWCNT@AFI host-guest material. Moreover, it was worth noting that multiple bound state soliton pulses also exhibited certain regularities, adding another dimension to their unique characteristics. We found that the central wavelength had always been around 1556 nm and did not affect the repetition rate or output power. Figure 10a displayed the output repetition rate of 29.24 MHz (period τ = 34.43 ns), which was determined by the cavity length of 7.05 m, including a gain fiber (PM-ESF) length of 1.2 m. The relationship between cavity length and repetition frequency was also consistent with Equation (3):

(3) $$f=\frac{c}{nL}$$

where *f* represents the repetition rate; *c* is the speed of light in vacuum; *n* is the fiber refractive index coefficient (approximately 1.46); and *L* is the cavity length.

In order to verify the stability of the bound state soliton pulse, we used the spectrum analyzer Keysight N9020B via a 15 GHz photodetector to measure its radio frequency (RF) spectrum. As shown in Figure 10b, the signal-to-noise ratio (SNR) was measured as 81 dB at a resolution of 2 kHz, and the repetition rate of the pulse was 29.24 MHz, corresponding to the image in the oscilloscope. In the inset of Figure 10b, we observed that the SNR remained stable without significant fluctuations over the 1 GHz span range, demonstrating good time-domain stability. We also found the same phenomenon in the spectrogram. The spectral evolution diagram is shown in Figure 10c. During our experiment, we conducted continuous recordings of the output sequence for two consecutive days, with measurements taken at 12 h intervals. Throughout this observation period, we consistently observed perfectly symmetrical spectra, indicating the stability of the mode-locked pulse. The pulse remained in a tightly bound state without any significant deviations or disruptions. This further highlighted the robustness and reliability of the experimental setup.

**Figure 11:**
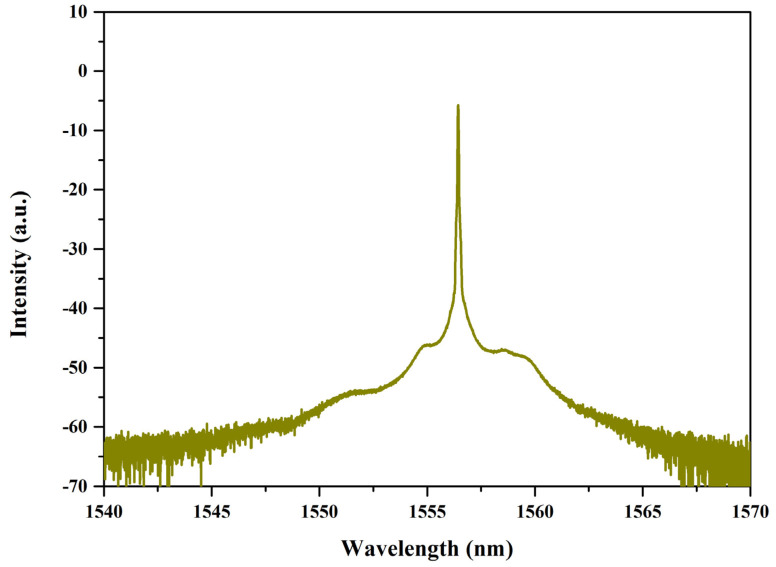
Spectrum of the all-PM optical path without the SWCNT@AFI SA (center wavelength 1556.44 nm).

In addition, we considered the possibility of self-phase modulation in the polarization maintaining optical path, and in order to eliminate this effect, we removed the SA from the fiber optic jumper end face. We found that only continuous light could appear during the process of increasing power (Figure 11), which confirmed the role of SA.

## 4. Conclusions

In this experiment, we successfully expanded the application of our self-made SWCNT@AFI SA to a new field and developed a simple method to generate multiple bound state soliton pulses. Moreover, the discovery of multiple bound state pulse states enriched our understanding of mode-locked phenomena. These findings served as an experimental foundation for further exploration of the mode-locking mechanism and the intricate interactions among bound state solitons. Finally, we verified the stability of our all-PM fiber laser, including the high SNR (frequency domain stability) and time-domain stability. Combined with the advantages of the strong anti-interference ability of PM fibers, it was expected to serve as a stable seed source for high-power lasers or amplification lasers and also contribute to the industrial production of laboratory results. Through the strong interaction between tight bound state solitons, the stability of the output laser was greatly improved, which had important research value in the fields of biomedicine, optical fiber communication, and high-resolution optics.

## Data Availability

All the data presented in this study are available in this article.
